# Cigarette Smoke-Induced Emphysema and Pulmonary Hypertension Can Be Prevented by Phosphodiesterase 4 and 5 Inhibition in Mice

**DOI:** 10.1371/journal.pone.0129327

**Published:** 2015-06-09

**Authors:** Michael Seimetz, Nirmal Parajuli, Alexandra Pichl, Mariola Bednorz, Hossein Ardeschir Ghofrani, Ralph Theo Schermuly, Werner Seeger, Friedrich Grimminger, Norbert Weissmann

**Affiliations:** Universities of Giessen and Marburg Lung Center (UGMLC), Excellence Cluster Cardio-Pulmonary System (ECCPS), Member of the German Center for Lung Research (DZL), Giessen, Germany; Indiana University, UNITED STATES

## Abstract

**Rationale:**

Chronic obstructive pulmonary disease (COPD) is a widespread disease, with no curative therapies available. Recent findings suggest a key role of NO and sGC-cGMP signaling for the pathogenesis of the disease. Previous data suggest a downregulation/inactivation of the cGMP producing soluble guanylate cyclase, and sGC stimulation prevented cigarette smoke-induced emphysema and pulmonary hypertension (PH) in mice. We thus aimed to investigate if the inhibition of the cGMP degrading phosphodiesterase (PDE)5 has similar effects. Results were compared to the effects of a PDE 4 inhibitor (cAMP elevating) and a combination of both.

**Methods:**

C57BL6/J mice were chronically exposed to cigarette smoke and in parallel either treated with Tadalafil (PDE5 inhibitor), Piclamilast (PDE4 inhibitor) or both. Functional measurements (lung compliance, hemodynamics) and structural investigations (alveolar and vascular morphometry) as well as the heart ratio were determined after 6 months of tobacco smoke exposure. In addition, the number of alveolar macrophages in the respective lungs was counted.

**Results:**

Preventive treatment with Tadalafil, Piclamilast or a combination of both almost completely prevented the development of emphysema, the increase in lung compliance, tidal volume, structural remodeling of the lung vasculature, right ventricular systolic pressure, and right ventricular hypertrophy induced by cigarette smoke exposure. Single, but not combination treatment prevented or reduced smoke-induced increase in alveolar macrophages.

**Conclusion:**

Cigarette smoke-induced emphysema and PH could be prevented by inhibition of the phosphodiesterases 4 and 5 in mice.

## Introduction

Chronic obstructive pulmonary disease (COPD) is a collective term for chronic bronchitis and emphysema and is one of the major causes of death worldwide [1]. On the one hand, airway inflammation and remodeling represent characteristic features. On the other hand, there is destruction of the elastic architecture of the lung which leads to enlargement of distal airspaces, causing emphysema [2]. In addition, COPD/emphysema is increasingly viewed as a systemic disease, involving skeletal muscle wasting, diaphragmatic dysfunction, and systemic inflammation [3]. Influx of inflammatory cells, imbalance of proteolytic and anti-proteolytic activity, increased oxidative stress with the rise in number of apoptotic cells and decreased proliferation might be important events underlying COPD [4–6].

Recent observations suggest an essential role of endothelial dysfunction and pulmonary hypertension (PH) for the development of COPD [7]. In animal models, cigarette smoke-induced emphysema was accompanied by pulmonary vascular remodeling and PH [8–11], and we and others could recently show that such vascular alterations even preceded alveolar destruction [8, 9]. The observations in animal models are supported by human studies showing that vascular remodeling can already occur in smokers without COPD [12, 13]. Moreover, the involvement of the pulmonary vasculature in COPD is reflected by the fact that up to 70% of COPD patients also suffer from PH [14].

Our own previous studies investigating nitric oxide (NO) as well as sGC-cGMP signaling revealed that 1) an inhibition of the NO-producing enzyme inducible NO synthase (iNOS) [9] and 2) a stimulation of the soluble guanylate cyclase (sGC) [15] prevented cigarette smoke-induced emphysema and PH. Furthermore, a curative approach using an iNOS inhibitor in diseased animals resulted in lung regeneration within three months [9]. Functionally essential sGC subunits have been shown to be downregulated upon smoke exposure in mice and in human COPD, suggesting a key role of cyclic guanosine monophosphate (cGMP) reduction as a driver of smoke-induced lung emphysema and PH [15]. Against this background the aim of the present study was to test the hypothesis that, similar to cGMP enrichment by sGC stimulation, inhibition (using Tadalafil) of the downstream cGMP degrading enzyme, phosphodiesterase (PDE)5, would prevent cigarette smoke-induced emphysema and PH in mice as well. For comparison we used a specific PDE4 inhibitor, Piclamilast [16], to assess the effects of a cAMP elevating agent. A combination of both inhibitors should reveal potential synergistic effects, possibly needed if single PDE inhibitor treatment would have no or only partially preventive effects. Despite the fact that PDE5 inhibitors have already been used in clinical trials in COPD patients with and without PH [17–25], this study provides the thus far missing data of long term treatment with PDE inhibitors and assesses detailed effects on alveolar and vascular structure which was not possible in the respective human studies.

## Methods

All experiments were performed according to institutional guidelines complying with national and international regulations. The study was approved by the Regierungspräsidium Giessen (Hessen, Germany). All analyses/exposures were done in a blinded, randomized fashion. For the study design sample size estimation was performed prior to the experiments.

### Exposure to smoke

Male C57BL6/J mice, body weight (19 to 20 g) were purchased from Charles River Deutschland, Sulzfeld, Germany. Mice were divided randomly into five groups (10 animals, each). Group 1: healthy control (no smoke exposure); group 2: control (Placebo [solvent application], smoke exposure), group 3: Piclamilast treatment (10 mg/kg body weight, smoke exposure); group 4: Tadalafil (10 mg/kg body weight, smoke exposure) and group 5: combination treatment with Piclamilast and Tadalafil (10 mg/kg body weight each, smoke exposure). The condition of the animals was monitored daily prior to treatment. Smoke challenged animals were whole body exposed [9] to tobacco smoke of 3R4F cigarettes (Kentucky Tobacco Research and Development Center, USA) at a particle concentration of 140 mg/m^3^ for 6 h/day, 5 days/week for a period of 6 months. 200 μl of solutions of Piclamilast and Tadalafil were freshly prepared and suspended in 4.0% methocel and 1.3% polyethyleneglycol via an Ultraturrax (IKA Staufen, Germany). To avoid foam formation, two drops of Antifoam C (Sigma-Aldrich) were added. The suspension was applied by gavage daily. After six months, functional parameters were assessed and lungs were fixed for morphometric analyses. Missing data are due to technical problems (e.g., catheter placement).

### Lung function tests

Animals were anaesthetized with ketamine (60 mg/kg body weight) and xylazine (10 mg/kg body weight) intraperitoneally and anticoagulated with heparin (1000 U/kg). The trachea was cannulated, and the lungs were ventilated with room air at a tidal volume of 200 μl and a frequency of 150 breaths per minute. Animals were maintained at physiological temperature throughout the experiment. A tracheal cannula was connected to a pneumotachometer (Hugo Sachs GmbH, Germany) and the tidal volume, resistance and dynamic compliance were evaluated using the HSE PULMODYN software (Hugo Sachs Electronics, March-Hugstetten, Germany) as described previously [9].

### *In vivo* hemodynamics

Animals were anaesthetized with ketamine (60 mg/kg body weight) and xylazine (10 mg/kg body weight) intraperitoneally and anticoagulated with heparin (1000 U/kg). The trachea was cannulated, and the lungs were ventilated with room air at a tidal volume of 200 μl and a frequency of 150 breaths per minute. Animals were maintained at physiological temperature throughout the experiment. Right ventricular systolic pressure (RVSP) was measured by inserting a PE-80 tube into the right ventricle via the right jugular vein and the systemic arterial pressure was determined by carotid artery catheterization as described previously [15, 26].

### Fixation of the lung

Lungs were flushed blood-free with saline via the pulmonary artery. For morphometry, left lungs were fixed with 4.5% paraformaldehyde in phosphate-buffered saline (pH 7.0) via the trachea at a pressure of 12 cm H_2_O and via the pulmonary artery at a vascular pressure of 22 cm H_2_O. Investigations were performed from 3 μm sections of paraffin embedded lungs.

### Alveolar morphometry

The development of emphysema was determined from lung sections stained with hematoxylin and eosin as described previously [27] and measured by quantitative morphometry (parameters: mean linear intercept, air space, and septal wall thickness [9]).

### Vascular morphometry

The degree of muscularization was determined from stained lung sections using antibodies against α-smooth muscle actin and von Willebrand factor as described before [9, 28]. Small (20–70 μm outer diameter), medium (70–150 μm outer diameter) and large (>150 μm outer diameter) vessels were classified as non-muscular (no smooth muscle cells detectable with actin staining), partially muscularized (at least one smooth muscle cell up to 75% of circumference with actin staining), and fully muscularized (>75% of circumference with actin staining). Vascular lumen area was determined from the same vessels analyzed for the degree of muscularization and is given as mean lumen area.

### Right ventricular hypertrophy

Right ventricular hypertrophy was quantified by the ratio of the right ventricular (RV) and the left ventricular + septum (S) mass. Hearts were removed directly after lung fixation and the RV was dissected from the LV+S. Values were determined from dried heart tissue [15].

### Quantification of the number of alveolar macrophages in lung sections

Macrophage counting was performed in 3-μm sections from paraffin-embedded lungs. After heating at 58°C, lung sections were deparaffinized in xylene and rehydrated. The endogenous peroxidase activity was quenched with 15% (v/v) H_2_O_2_ in methanol. For staining, a 1:10 dilution of macrophage marker F4/80 antigen-specific antibody (rat monoclonal to F4/80 [ab6640], Abcam, Cambridge, UK) was used. Immune complexes were visualized with a peroxidase-conjugated secondary antibody and NovaRED Peroxidase (HRP) Substrate Kit (Vector labs, LINARIS, Wertheim-Bettingen, Germany) and Hematoxylin solution was used for counterstaining of the sections. The quantification of macrophages and measurement of the lung areas were carried out microscopically using a Qwin macro program from Leica (Wetzlar, Germany). For each lung, the total number of macrophages per lung section was counted. Data were calculated as amount of macrophages/mm^2^.

### Data analysis

All data are given as mean ± SEM. Differences between more than two groups were assessed by ANOVA followed by Student-Newman-Keuls or Dunnett’s post hoc test. P values <0.05 were considered as significant. All statistical calculations were performed using GraphPad Prism software.

## Results

### Effect of Piclamilast and/or Tadalafil on cigarette smoke-induced lung emphysema development

**Fig 1:**
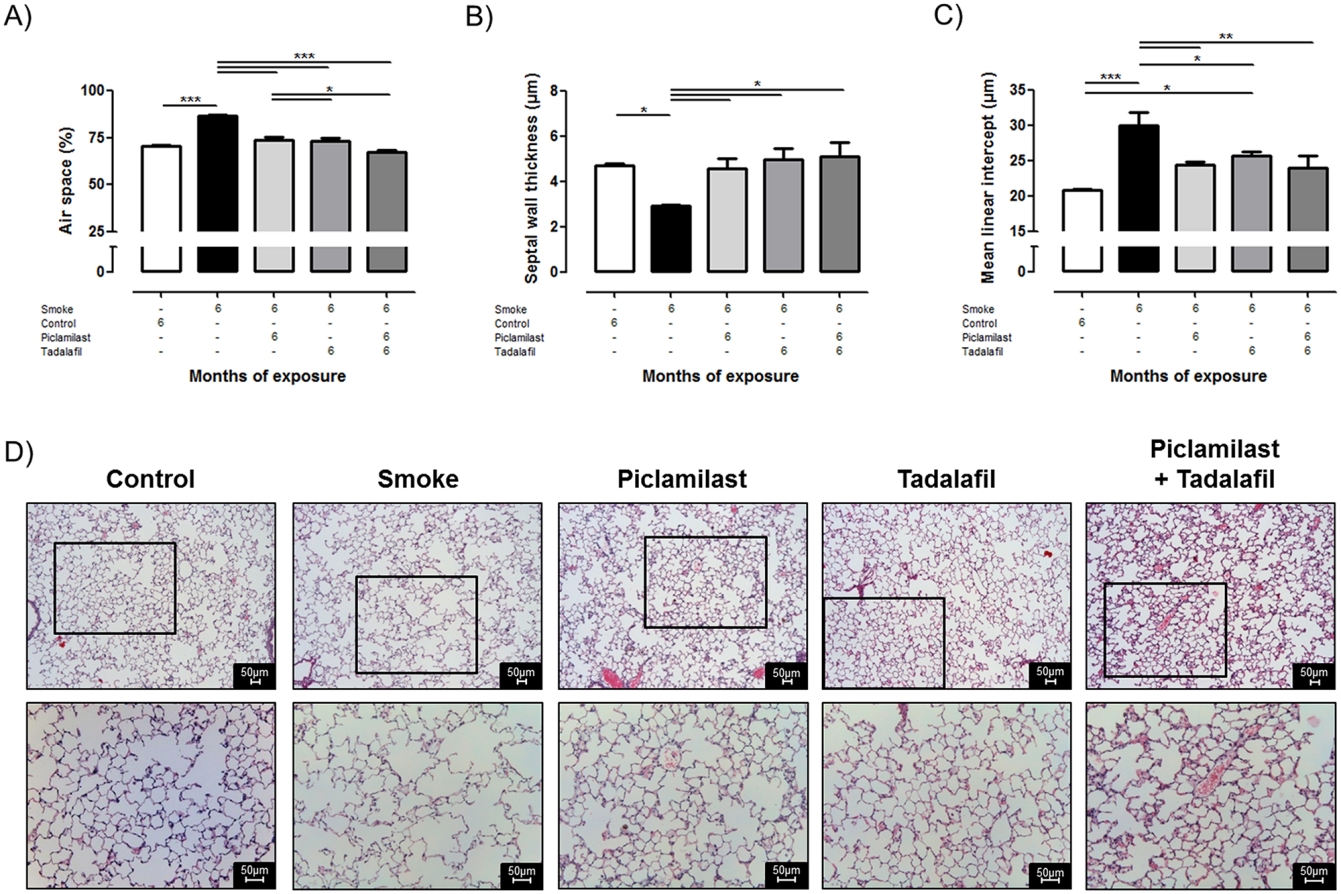
Effect of Piclamilast and/or Tadalafil on cigarette smoke-induced lung emphysema development assessed by structural parameters. Mice were exposed to cigarette smoke for 6 months and treated in parallel with Piclamilast (10 mg/kg body weight/day) and/or Tadalafil (10 mg/kg body weight/day). Time matched controls received solvent only. **(A)** Air space, **(B)** septal wall thickness and **(C)** mean linear intercept. **(D)** Representative histology from mice lung sections stained with hematoxylin and eosin. Data are given as mean ± SEM from n = 5–6, each. Bars indicate significant differences using ANOVA followed by Student-Newman-Keuls post hoc test (*P<0.05;** *P*<0.01; ***P<0.001).

**Fig 2:**
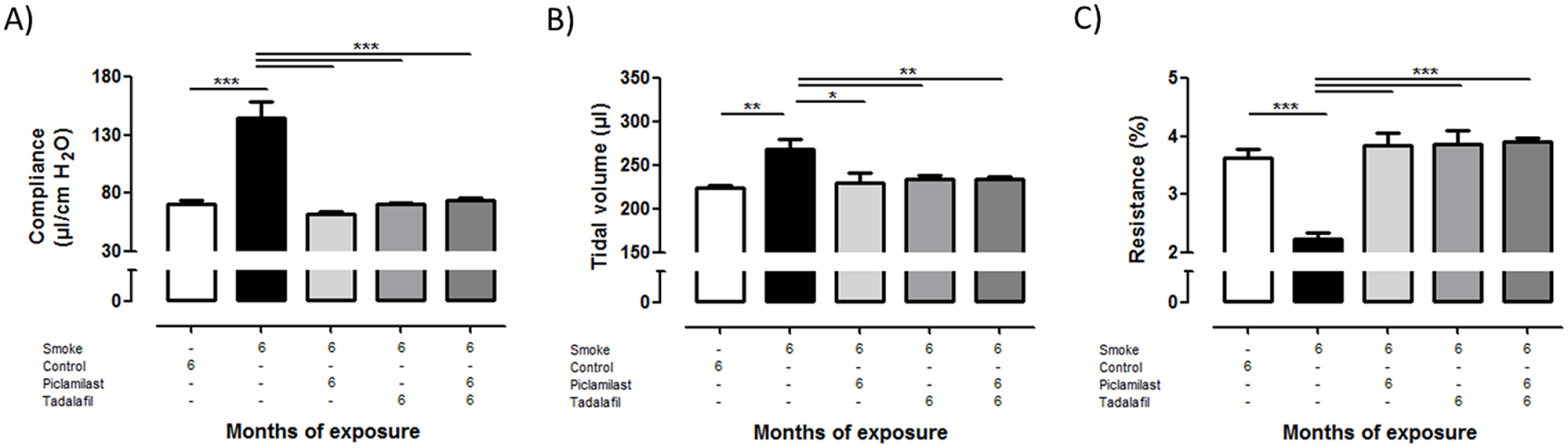
Effect of Piclamilast and/or Tadalafil on cigarette smoke-induced lung emphysema development assessed by *in vivo* lung functional parameters. Mice were exposed to cigarette smoke for 6 months and treated in parallel with Piclamilast (10 mg/kg body weight/day) and/or Tadalafil (10 mg/kg body weight/day). Time matched controls received solvent only. **(A)** dynamic lung compliance, **(B)** tidal volume and **(C)** airway resistance. Data are given as mean ± SEM from n = 6–9, each. Bars indicate significant differences using ANOVA followed by Student-Newman-Keuls post hoc test (* *P*<0.05**; *P*<0.01; ***P<0.001).

Tobacco smoke-exposure of mice for 6 months resulted in development of lung emphysema, quantified by an increase in airspace, mean linear intercept, and a decrease in septal wall thickness (Fig 1). These structural alterations were reflected by respective changes in lung compliance, tidal volume as well as airway resistance *in vivo* (Fig 2). Treatment with either Piclamilast, Tadalafil or a combination of both in parallel with smoke exposure prevented the development of lung emphysema as evident from the structural as well as the functional parameters which were not different from untreated non-exposed control mice (Figs 1 and 2). No differences between Piclamilast, Tadalafil or the combination therapy could be detected.

### Effect of Piclamilast and/or Tadalafil treatment on smoke-induced pulmonary hypertension, pulmonary vascular remodeling, and right heart hypertrophy

**Fig 3:**
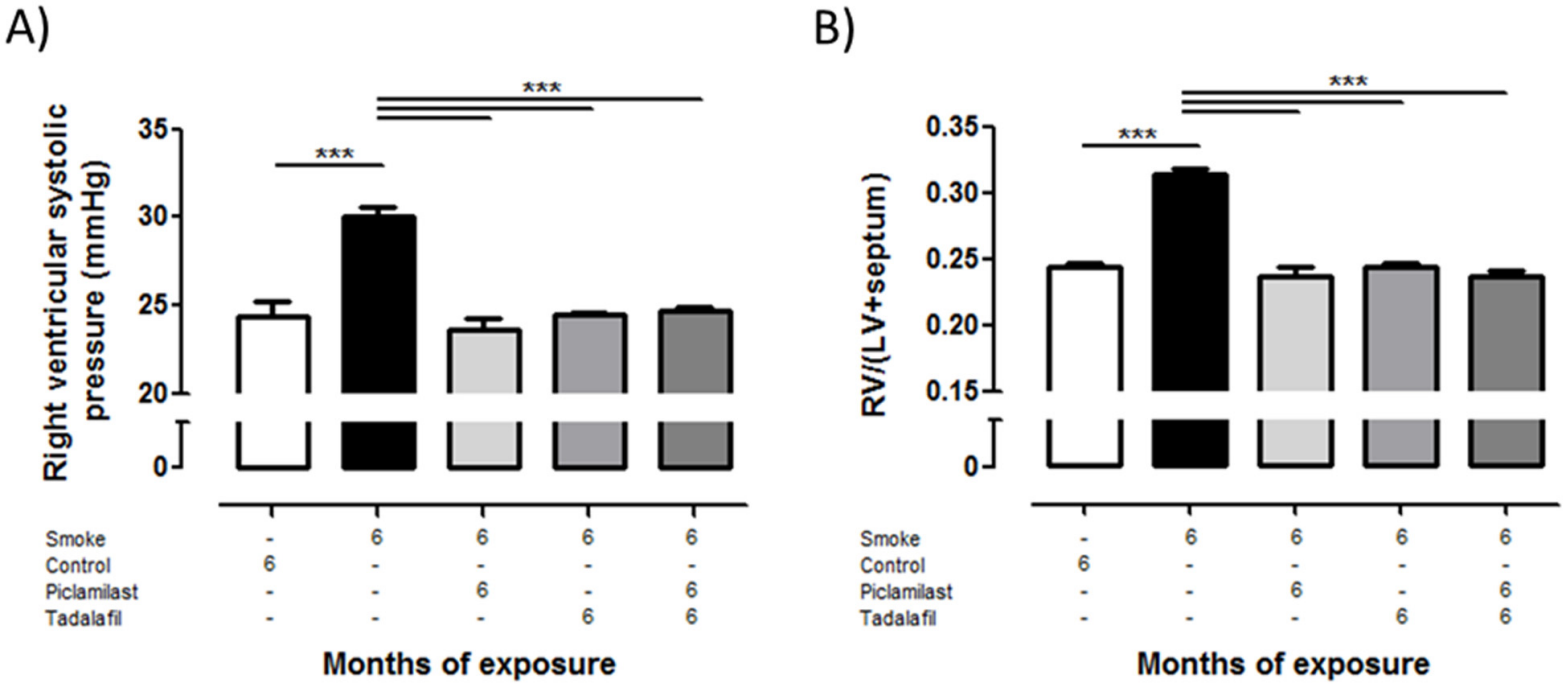
Effect of Piclamilast and/or Tadalafil on cigarette smoke-induced pulmonary hypertension in mice assessed by functional parameters. Mice were exposed to cigarette smoke for 6 months and treated in parallel with Piclamilast (10 mg/kg body weight/day) and/or Tadalafil (10 mg/kg body weight/day). Time matched controls received solvent only. **(A)** Right ventricular systolic pressure quantified by right heart catheterization in anesthetized animals. **(B)** Right heart hypertrophy, given as the ratio of right ventricular (RV) mass to left ventricular plus septum (LV+S) mass, from dried heart tissue; left ventricular weights did not differ between the different groups. Data are given as mean ± SEM from n = 5–8, each. Bars indicate significant differences using ANOVA followed by Student-Newman-Keuls post hoc test (**P<0.01; ***P<0.001).

**Fig 4:**
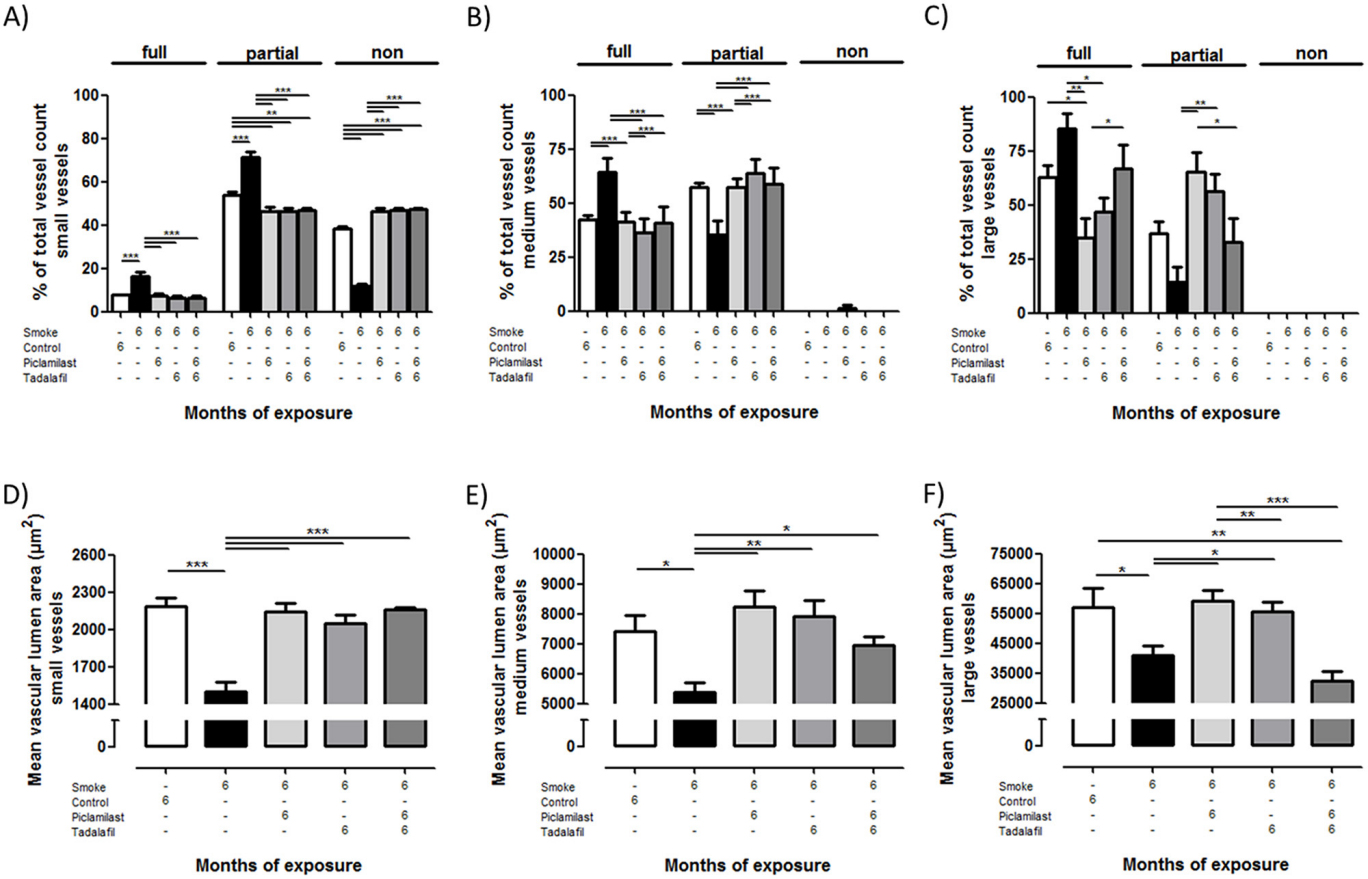
Effect of Piclamilast and/or Tadalafil on cigarette smoke-induced pulmonary vascular remodeling in mice assessed by structural parameters. Mice were exposed to cigarette smoke for 6 months and treated in parallel with Piclamilast (10 mg/kg body weight/day) and/or Tadalafil (10 mg/kg body weight/day). Time matched controls received solvent only. **(A-C)** Degree of muscularization of **(A)** small pulmonary arteries (outer diameter 20–70 μm), **(B)** medium vessels (outer diameter >70 to 150 μm), and **(C)** large vessel (outer diameter >150 μm), as a percentage of total vessel count for fully muscularized (full), partially muscularized (partial), and non-muscularized (non) vessels. **(D-F)** Mean vascular lumen area of **(D)** small pulmonary vessels (outer diameter 20–70 μm), **(E)** medium vessels (outer diameter >70 to 150 μm), and **(F)** large vessel (outer diameter >150 μm). Data are given as mean ± SEM from n = 6, each. Bars indicate significant differences using ANOVA followed by Student-Newman-Keuls post hoc test (*P<0.05; **P<0.01; ***P<0.001).

**Fig 5:**
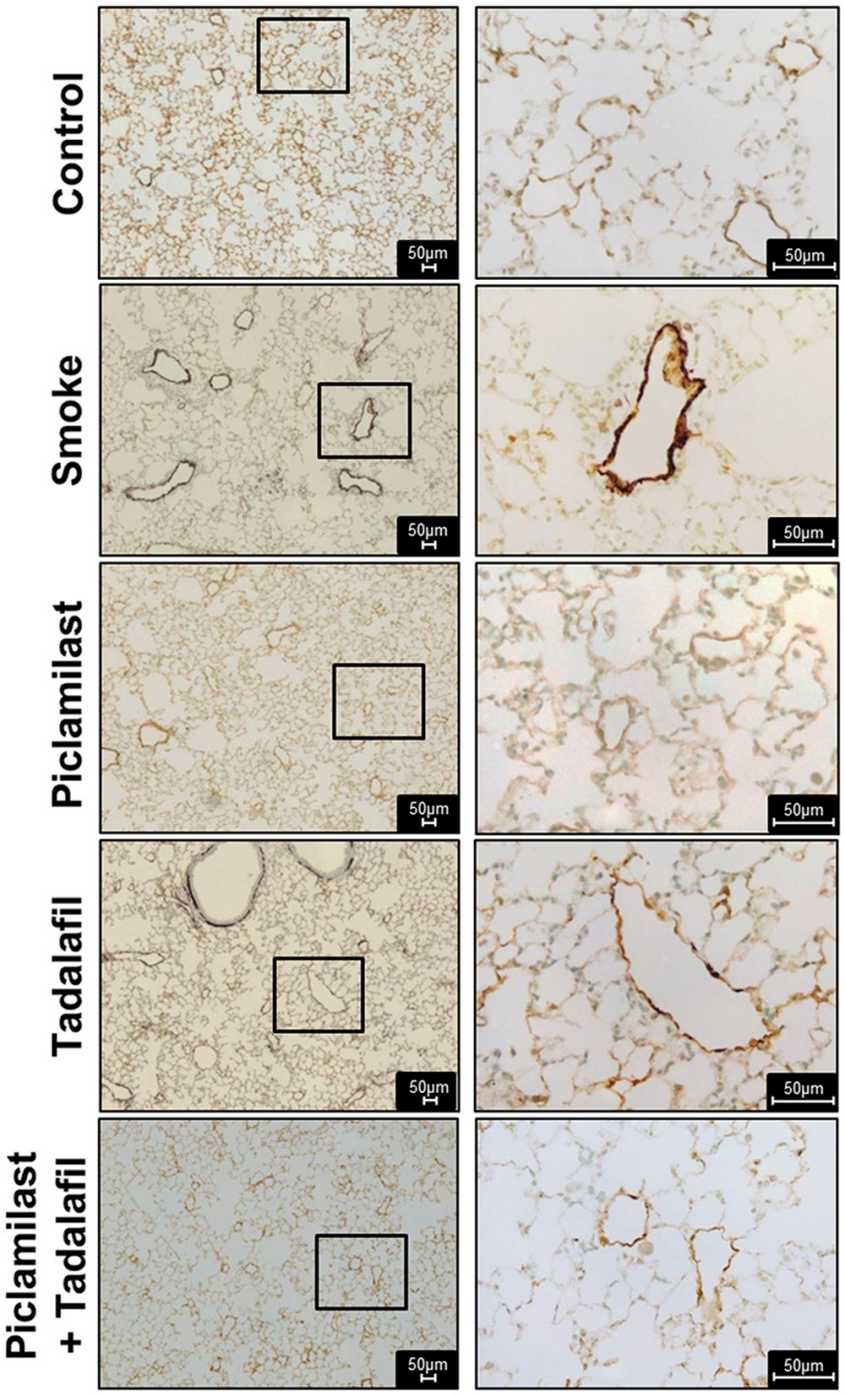
Effect of Piclamilast and/or Tadalafil on cigarette smoke-induced pulmonary vascular remodeling in mice—histology. Representative histology from lung sections stained with antibodies against α-smooth muscle actin and von Willebrand factor.

Tobacco smoke-exposed mice developed pulmonary hypertension as determined by increased right ventricular systolic pressure (Fig 3A). Moreover, smoke exposure resulted in vascular remodeling reflected by an increased degree of muscularization in all categories of vessel diameters assessed (Fig 4A–4C) and decreased the vascular lumen area in all vessels (Fig 4D–4F). Furthermore, the right ventricle was augmented upon chronic smoke exposure shown by the ratio of RV/LV+S (Fig 3B) compared to control mice. Treatment of mice with Piclamilast and/or Tadalafil resulted in a complete protection against the development of pulmonary hypertension as displayed in Figs 3–5. Of note, the lumen of large vessels was significantly decreased after the combination therapy compared to both single treatments (Fig 4F).

### Systemic effects of smoke exposure and inhibition of phosphodiesterases

**Fig 6:**
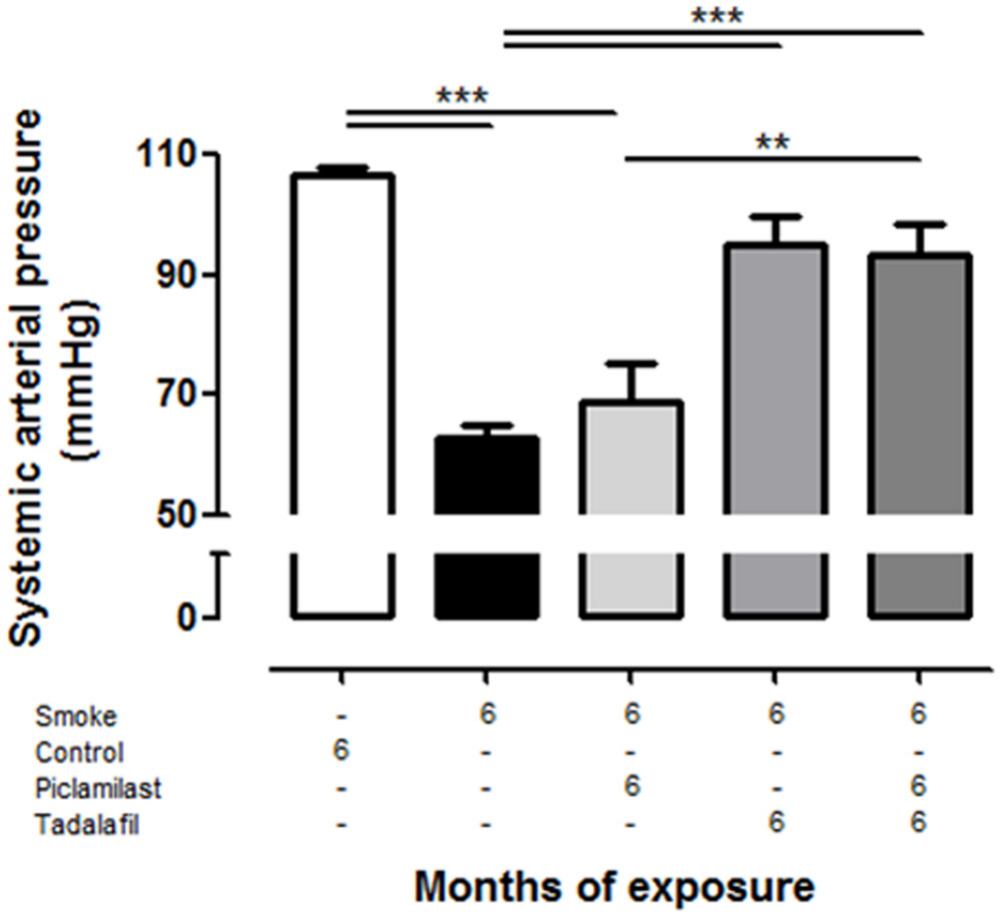
Systemic effects of smoke exposure and treatment with Piclamilast and/or Tadalafil in mice. Mice were exposed to cigarette smoke for 6 months and treated in parallel with Piclamilast (10 mg/kg body weight/day) and/or Tadalafil (10 mg/kg body weight/day). Time matched controls were received solvent only. Systemic arterial pressure determined by *A*. *carotis* catheterization and quantified in anesthetized animals. Data are given as mean ± SEM from n = 5–8, each. Bars indicate significant differences using ANOVA followed by Student-Newman-Keuls post hoc test (**P<0.01; ***P<0.001).

To determine possible systemic consequences of right heart hypertrophy and PH seen in smoke-exposed mice, systemic arterial pressure (SAP) was measured via *A*. *carotis*. These data revealed a significant decrease of SAP in untreated smoke-exposed mice compared to control mice which was prevented by Tadalafil and the combination therapy, but not by the sole application of Piclamilast (Fig 6).

### Anti-inflammatory effect of Piclamilast and/or Tadalafil

**Fig 7:**
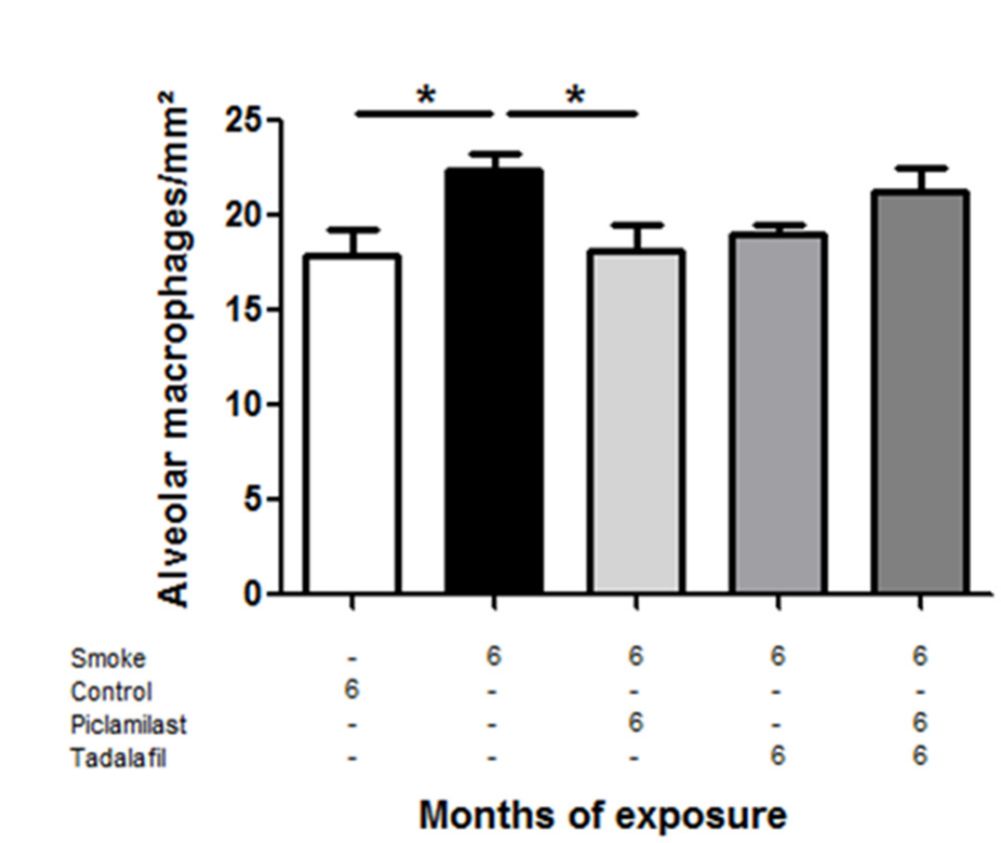
Effect of smoke exposure, Piclamilast and/or Tadalafil treatment on the number of alveolar macrophages in mice. Lung paraffin sections were stained with a macrophage-specific antibody (F4/80) and respective alveolar macrophages from whole sections were counted and calculated as number of macrophages/mm^2^. Data are given as mean ± SEM from n = 5, each. Bars indicate significant differences using ANOVA with Dunnett’s post hoc test (*P<0.05).

Based on already described anti-inflammatory action of PDE4 and -5 inhibitors, we counted the number of alveolar macrophages as an indicator of inflammatory response in lung sections derived from our smoke-exposed and control mice. Tobacco smoke-exposed mice showed a significant increase of alveolar macrophages per mm^2^, which was significantly attenuated by Piclamilast. Tadalafil treatment showed a trend toward reduction, and interestingly, no such effect was evident for the combination therapy (Fig 7).

## Discussion

The present study answers the question of whether an enrichment of the second messengers cGMP and cAMP by PDE inhibition could potentially be used for long-term treatment of cigarette smoke-induced emphysema and PH. Indeed, we demonstrated in a long-term mouse study that the PDE5 inhibitor Tadalafil and the PDE4 inhibitor Piclamilast as well as a combination of both prevented smoke-induced emphysema and pulmonary hypertension. Synergistic effects of the combination therapy could not be observed since single therapies have already had strong effects, almost to the level of unexposed control animals. The decline of systemic arterial pressure (SAP) caused by cigarette smoke exposure could be prevented by Tadalafil as well as by the combination of Tadalafil and Piclamilast but not by Piclamilast treatment alone. Although much is known about PDE inhibitors in lung diseases and such inhibitors are used in the clinic, human studies do not allow one to assess effects of PDE inhibitors on the alveolar and vascular structure. These drawbacks can be overcome by experimental studies in mice as in our investigation.

Although with these experiments we only investigated a preventive and not a curative treatment, any means to stop the progression of structural and functional deterioration in COPD would be of value if transferable to the human situation. Furthermore, it is conceivable that even longer-term treatment with these agents could cure the disease, the holy grail of lung emphysema treatment, a possibility which warrants further investigation.

Previous data suggest an important role of NO as well as sGC-cGMP signaling in the physiology and pathophysiology of pulmonary vasculature [29] as well as in airway disease [15]. This system controls many vascular functions such as vascular tone, homeostasis, structure and remodeling [30, 31]. An imbalance of this system may result in PH and other pulmonary diseases [26, 32]. cGMP is not only an important regulator of short-term changes in smooth muscle tone but also in long-term responses to chronic pro-proliferative signals [33]. Nitric oxide (NO), produced by NO synthases (NOS), can activate sGC, which subsequently increases the levels of cGMP [34]. However, iNOS upregulation has been shown to cause emphysema in mice, which may be explained by iNOS-dependent sGC oxidation and inactivation [9].

In line with the previous finding that sGC stimulation by Riociguat can prevent from smoke-induced PH and emphysema in mice and guinea pigs [15], the PDE5 inhibitor Tadalafil also prevented such smoke-induced changes as demonstrated in the present study. PDE5 is highly specific for cGMP hydrolysis and is thought to be the most active cGMP hydrolyzing PDE in smooth muscle cells under basal conditions [33]. Although the mechanisms were not investigated in our study, it is well known that nitric oxide, atrial natriuretic peptide (ANP) and several other vasodilators use cGMP to regulate the vascular tone [33]. cGMP activates the cGMP-dependent protein kinase (PKG). An increase in cGMP results in a decrease of the intracellular Ca^2+^ concentration which leads to vasodilation [33]. The cGMP effects on contraction in smooth muscle seem to be specifically mediated by PKG but not by cAMP-dependent protein kinase (PKA). This finding was validated using PKG-1-deficient mice where cGMP-induced relaxation of aortic smooth muscle was completely abolished, whereas cAMP-dependent relaxation was not affected [35]. Phosphorylation of specific PKA substrates such as regulatory myosin-binding subunit of myosin phosphatase [36], IP_3_ receptor associated cGMP kinase substrate (IRAG) [37] and calcium-activated maxi K^+^ (BK_Ca_) channels [38], contribute to the reduction of intracellular calcium which finally causes decreased muscular tone [39]. Both cGMP and cAMP modulate acute smooth muscle relaxation, mainly through the reduction of intracellular calcium or activation of myosin phosphatase [33]. In addition to vascular tone regulation, which can also affect vascular remodeling processes e.g., by altered shear stress, cGMP elevating therapies have previously been shown to protect the organism from increased proliferation underlying pulmonary vascular remodeling [26, 30, 32, 33]. Such effects may explain the protection from vascular remodeling and PH in our study. The protection from emphysema is in line with the concept that molecular pulmonary vascular alterations drive smoke-induced emphysema development [9]. Alternatively, the protection from emphysema by PDE5 inhibition could also be independent of its vascular effects. Since only the single therapies, but not the combination therapy, prevented vascular narrowing in large vessels, it can be speculated that a parallel inhibition of both PDEs has negative effects by inhibiting anti-proliferative pathways differentially regulated in large vessels compared to small vessels.

Interestingly, application of the PDE4 inhibitor which was shown to increase cAMP [40], had quite similar effects to those observed after PDE5 inhibition. This finding is again in line with the suggestion that pulmonary vascular alterations can drive emphysema development—cAMP-elevating therapies have been shown in different animal models to protect against non-smoke-induced forms of PH [41, 42] as well and prostanoid therapy is used for treatment of human PH [43, 44]. Although the pathways of generation and regulation of cAMP and cGMP are different, the final effects in terms of vasodilation, anti-inflammation, and anti-proliferation are quite similar and not always unrelated. For instance, it could be shown that PDE5 inhibitors reduce proliferation of bovine coronary artery SMCs via cGMP elevation and subsequent inhibition of PDE3, a cGMP-inhibitable cAMP-hydrolyzing PDE [45]. These data demonstrate the possibility of increasing cAMP in a cGMP-dependent manner. Potential additional therapeutic effects of the combination of Tadalafil and Piclamilast could not be evaluated in our study as single application of each inhibitor has already had a maximum effect. Thus, investigations determining possible additive effects must be performed with lower dosages of the inhibitors.

It could be demonstrated *in vitro* that the oxidative burst in sputum from asthma and COPD patients could be reduced by Piclamilast [46]. Such anti-oxidant, but also anti-apoptotic and anti-proliferative properties of Piclamilast were recently confirmed *in vitro* by Mata and colleagues [16]. Although not shown in the present study, these Piclamilast-dependent effects could also, at least in part, explain the prevention of emphysema and vascular remodeling in smoke-exposed mice upon PDE4 inhibition.

A limitation of our study is that we could not measure cAMP/cGMP levels in our mice directly when using lung homogenate. This issue can be related either 1) to methodological limitations of our cAMP and cGMP measurement approach or 2) cell type-specific elevation of cAMP and cGMP concentration, not being reflected by lung homogenate measurements. Also variations in drug preparation, dosing, and administration strategies may result in significant differences in drug levels and, therefore, target effects. However, it was previously shown that PDE 4 and 5 inhibitors including Tadalafil and Piclamilast can elevate cGMP and cAMP levels and have been used *in vivo* in rat, rabbit, guinea pig and mouse models [27, 42, 47–56].

The decrease in SAP upon smoke exposure can principally be explained by either a direct effect of tobacco smoke on the systemic circulation or an impaired left-ventricular function due to PH-associated reductions in pre-load and cardiac output [57]. Since treatment with the PDE5 inhibitor or a combination of PDE4 and 5 inhibitors prevented PH and its corresponding pre-load effects, the latter explanation does not seem valid. Instead the data suggest a direct effect of tobacco smoke on the systemic circulation. Interestingly, only the presence of the PDE5 inhibitor could prevent the systemic effects, suggesting that cAMP elevation alone does not counteract systemic hemodynamic effects of tobacco smoke.

Although clinical studies using Sildenafil reported no change in exercise capacity, quality of life and V/Q mismatch, long term data such as in our mouse study (when comparing the life-span of mice and humans) are not available for PDE5 inhibitors in lung emphysema. Concerning PDE4 inhibitors, Roflumilast has recently been approved as anti-inflammatory therapy for treatment of COPD. However, these clinical studies do not allow the assessment of long-term effects on alveolar and lung vascular structure.

A recent clinical study using Tadalafil in COPD patients demonstrated pulmonary vasodilation, but the exercise capacity and quality of life remained unchanged [23]. Although disappointing at first, limitations of this study were:”…no right heart catheterisation for patient selection and assessment of treatment response,...use of fairly modest indicators of pulmonary vascular disease for assessment of inclusion in the study…” (false positive diagnosis at inclusion). Of interest, the authors suggest higher effects of Tadalafil in COPD patients with severe PH. This suggestion is supported by a small randomized controlled clinical trial using Sildenafil treatment in patients with severe PH. It was reported that the PAP was decreased, associated with an increase in the 6-min walk distance [24]. Since Sildenafil improves exercise capacity and quality of life in PAH patients [58] and pulmonary hemodynamics at rest and during exercise in patients with COPD-associated PH, it seems that inhibition of PDE5 could be a reliable target for patients suffering from vascular alterations. This suggestion is supported by our observation that protection against vascular remodeling and PH in cigarette smoke-exposed mice was paralleled with prevention from emphysema development. Taken together, these data support the hypothesis of an essential role of the vasculature for the development of emphysema/COPD.

Based on the facts that PDE4 inhibitors suppress inflammatory processes in several cell types involved in COPD [59], inhibit cellular trafficking and microvascular leakage, produce reactive oxygen species, express cell adhesion molecules [60] and mediate lung smooth muscle relaxation, selective inhibitors have been developed for the treatment of COPD [61]. For instance, the PDE4 inhibitor Roflumilast has been used as anti-inflammatory therapy for the treatment of COPD. It reduces the numbers of neutrophils (35%) and eosinophils (50%) in sputum of COPD patients [62]. Along this line Piclamilast treatment significantly prevented the smoke-induced increase in the number of alveolar macrophages in our study. Interestingly, Tadalafil tended to have the same effect. However, no such tendency was observed for the combined treatment with Piclamilast and Tadalafil, questioning that the prevention from PH and emphysema was related to macrophage/inflammatory effects. Independently, several clinical studies have demonstrated that Roflumilast [63, 64] and Cilomilast [61] can improve lung function, quality of life and reduce the frequency of exacerbations. A beneficial effect of Piclamilast in sputum of COPD patients has been reported *in vitro* [46], and now in our animal model of cigarette smoke-induced emphysema and PH. However, a clinical trial using Piclamilast in COPD or COPD-PH patients has yet to be conducted.

In conclusion, our present data substantiate the recent suggestion, which was derived from investigations of guanylate cyclase stimulators, that sGC stimulation and possible downstream signaling via cGMP can, in long-term studies, prevent lung structural and functional deterioration induced by tobacco smoke. Comparison to the effects of a PDE4 inhibitor showed in addition that another, but mechanistically quite similar pathway, the AC-cAMP signaling, may mediate comparable protective mechanisms since the elevation not only of cGMP, but also cAMP resulted in protection from cigarette smoke-induced emphysema and PH.

## Supporting Information

S1 ARRIVE Checklist (PDF)
